# Radio(chemo)therapy in anaplastic thyroid cancer—high locoregional but low distant control rates—a monocentric analysis of a tertiary referral center

**DOI:** 10.1007/s00066-022-01943-0

**Published:** 2022-05-06

**Authors:** Matthias Schmied, Sebastian Lettmaier, Sabine Semrau, Maximilian Traxdorf, Konstantinos Mantsopoulos, Sarina K. Mueller, Heinrich Iro, Axel Denz, Robert Grützmann, Rainer Fietkau, Marlen Haderlein

**Affiliations:** 1 Department of Radiation Oncology, University Hospital of Erlangen, Friedrich-Alexander-University Erlangen-Nürnberg (FAU), Universitätsstr. 27, 91054 Erlangen, Germany; 2 Department of Otorhinolaryngology, Head and Neck Surgery, University Hospital of Erlangen, Friedrich-Alexander University Erlangen-Nürnberg (FAU), Waldstrasse 1, 91054 Erlangen, Germany; 3 Department of Surgery, University Hospital of Erlangen, Friedrich-Alexander-University Erlangen-Nürnberg (FAU), Krankenhausstrasse 12, 91054 Erlangen, Germany

**Keywords:** Anaplastic thyroid cancer, Radiotherapy, Chemotherapy, Radiation dose, Multimodal treatment

## Abstract

**Background:**

Anaplastic thyroid cancer (ATC) is a lethal disease with highly aggressive disease progression. This study analyses the influence of radio(chemo)therapy, R(C)T, on disease control, survival rates and predictors for survival.

**Patients and methods:**

A total of 33 patients with ATC, treated at a tertiary referral center between May 2001 and April 2020 were included. Univariate and multivariate analysis were used to investigate correlates of R(C)T and predictors on disease control and survival rates.

**Results:**

Median follow-up was 4 months. In UICC stage IVA and IVB median overall survival (OS) was 8 months, median progression-free survival (PFS) was 6 months. Patients with UICC stage IVA and IVB and patients being irradiated with a radiation dose of more than 60 Gy showed increased OS. Of these patients, 3 were alive and free from disease. All of them receiving cisplatin-based radiochemotherapy and a minimum radiation dose of 66 Gy. UICC stage IVC showed a median OS of 2.5 months and a median PFS of 1 month. Only 2 of 16 patients had local failure.

**Conclusion:**

Depending on UICC stage, RT with high radiation dose can lead to improved OS or at least higher locoregional control. A limiting factor is the high incidence of distant metastases; therefore modern systemic treatment options should be integrated into multimodal therapy concepts.

## Background

Anaplastic thyroid cancer (ATC) is an orphan disease accounting for only 2–5% of all thyroid cancers but for about 50% of all thyroid cancer deaths. Anaplastic thyroid cancer is described as a lethal disease with overall survival (OS) rates of usually less than 20% after 1 year as a result of its highly aggressive disease progression [1–4]. About 50% of all patients with ATC show distant metastases at initial diagnosis. Moreover ATC usually shows an aggressive local growth pattern with infiltration of neighboring organs and the large vascular structures leading to local symptoms like dyspnea, dysphagia among others [5]. The aim of multimodal therapeutic approaches including surgery, radiotherapy and systemic therapy is to achieve local control and thereby increase progression-free survival (PFS). Retrospective database analysis shows a survival benefit from radiotherapy [6] in patients with ATC and a survival benefit by adding chemotherapy to radiotherapy [1, 3, 7].

In current guidelines [8], radiotherapy is recommended in patients with stage IVA and IVB disease and possibly in patients with stage IVC cancer. However, detailed recommendations on radiotherapy concepts are missing. In this retrospective study we investigated disease control, survival rates and relevant predictors for survival in patients with ATC after radio(chemo)therapy (R[C]T), treated at a tertiary referral center.

## Patients and methods

All patients with anaplastic thyroid carcinoma treated at the department of radiation oncology at the University Hospital of Erlangen between May 2001 and April 2020 were included in this analysis. Records of 33 patients were studied. All tumors were classified according to the TNM classification system from 2017, 8th edition. For detailed patient characteristics see Table 1.

**Table 1:** Patient characteristics (*n* = 33)

Characteristics	No. of patients	%
*Sex*		
Female	22	67.7
Male	11	33.3
*Age at diagnosis, years*		
Median	67	–
Range	30–93	–
*Nicotine abuse*		
Yes	5	15.2
Never	22	66.7
Earlier	4	12.1
Unknown	2	6.1
*UICC*		
IVA	2	6.1
IVB	15	45.5
IVC	16	48.5
*Therapy*		
Surgery followed by radiotherapy (RT)	3	9.1
Surgery followed by radiochemotherapy (RCT)	9	27.3
Surgery, palliative RT	7	21.2
Surgery, palliative RCT	1	3.0
Definitive RCT	2	6.1
Definitive RT	1	3.0
Palliative RT	3	9.1
Palliative RCT	6	18.2
Best supportive care (BSC)	1	3.0
*Biopsy-exceeding surgery*		
Yes	20	60.6
No	13	39.4
*Resection margin*		
Complete resection (R0)	5	15.2
Microscopic residual disease (R1)	6	18.2
Macroscopic residual disease (R2)	8	24.2
Unclear resection margin (Rx)	1	3.0
No surgery performed	13	39.4
*Combined RCT*		
Yes	15	45.5
No	18	54.5
Applicated chemotherapy (CT)		
No CT	15	45.5
Cisplatin	14	42.4
Carboplatin	1	3.0
Cisplatin, paclitaxel	2	6.1
Adriamycin	1	3.0
*Applicated radiation single dose (Gy)*		
Median	2.6	–
Range	1.8–3.0	–
*Applicated radiation dose (Gy)*		
Median	45.5	–
Range	13.2–70.2	–

### Statistics

The statistical analysis was carried out using SPSS version 26 (IBM, Armonk, NY, USA).

OS and DFS were calculated from the date of a patient’s initial tumor diagnosis (defined as the date of biopsy or surgical resection) until the date of a patient’s death or the last available follow-up using the Kaplan–Meier method.

For statistical analysis radiation doses were calculated as EQD2 assuming an alpha/beta value of 10.

Considering the limited number of patients a dichotomized risk classification of the following factors was performed: gender, age (< 65 years vs. ≥ 65 years), nodal status (lymph node metastasis vs no lymph node metastasis), UICC stage (UICC A and B vs UICC C), resection status (R0/R1 vs R2/no resection), radiochemotherapy vs radiotherapy, applied radiation dose (<40 Gy vs ≥ 40 Gy; ≤ 60 Gy vs > 60 Gy), time interval between first diagnosis and start of radiotherapy (≤ 3 weeks vs > 3 weeks), surgery vs biopsy only. Risk factors were compared for OS using log rank test for univariate and Cox regression for multivariate analysis.

## Results

### All patients

Median follow-up was 4 months (range 1–86 months). UICC stage was as follows: UICC IVA: 2 patients (6.1%), IVB: 15 patients (45.5%), IVC: 16 patients (48.5%).

In all, 16 patients showed distant metastases at initial diagnosis. 12 patients were irradiated after surgery, 4 received definitive radiotherapy, 16 palliative radiotherapy (with or without prior surgery in the primary tumor region) and 1 patient died before the start of radiotherapy because of a rapidly progressive primary tumor with consecutive thrombosis extending to the right atrium (best supportive care).

A total of 18 patients were treated with concomitant chemotherapy: 17 patients received platinum-based chemotherapy while 1 patient received chemotherapy with adriamycin.

The prescribed radiation dose for curative treatment was as follows: median total dose: 65.4 Gy (range 33.0–70.2 Gy), median single dose: 2 Gy (range 1.8–3.0 Gy).

In case of palliative treatment, the prescribed radiation dose was as follows: median total dose: 37.5 Gy (range 13.2–56.8 Gy), median single dose: 3 Gy (range 2.0–3.0 Gy).

One patient died during radiotherapy because of progressive distant metastases. One patient with pulmonary metastases was lost to follow-up after premature termination of radiotherapy (due to patient wish). Three patients died shortly (within 10 weeks after end of RT) after the end of radiotherapy before the first follow-up visit and therefore the site of disease recurrence in these cases is not known with certainty. In these patients, disease in the local tumor region was controlled at the end of radiotherapy but patients had diffuse untreated distant metastases.

**Fig. 1:**
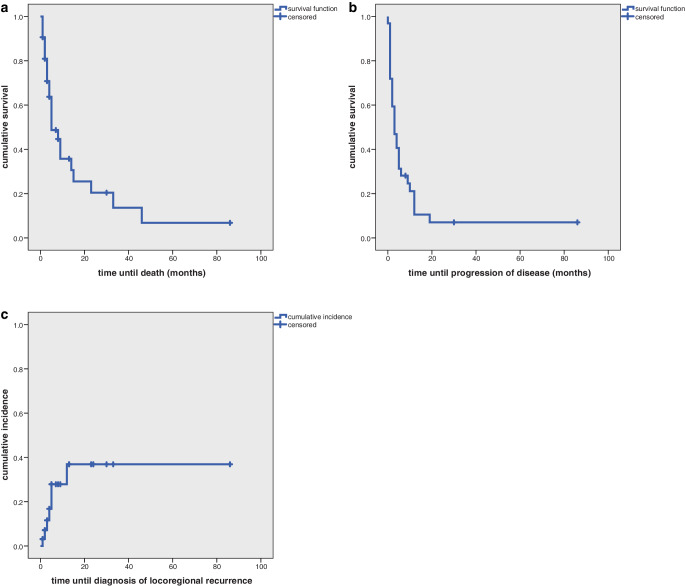
**a** Cumulative overall survival of all patients (*n* = 32). **b** Cumulative progression-free survival of all patients (*n* = 32). **c** Cumulative incidence of all patients (*n* = 32)

Median OS was 5 months (range 1–86 months) and median PFS was 3 months (range 0–86 months). Cumulative OS after 1 and 2 years was 35.7% and 20.4% (Fig. 1a) and cumulative PFS after 1 and 2 years was 10.5% and 7% (Fig. 1b).

Locoregional recurrences were detected in 7 patients. Cumulative incidence of locoregional recurrence was 36.9% after 1 and 2 years (Fig. 1c).

**Fig. 2:**
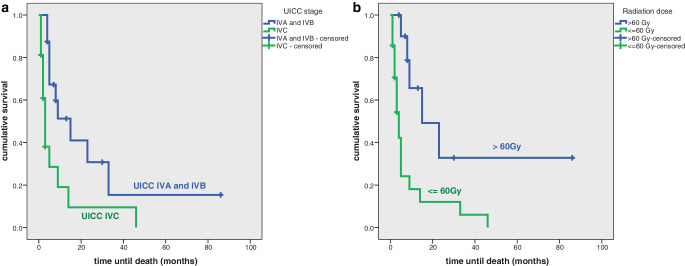
**a** Cumulative overall survival depending on UICC stage in all patients (*n* = 32). **b** Cumulative overall survival depending on applied radiation dose in all patients (*n* = 32)

Overall survival was significantly increased in patients with UICC stage IVA and IVB (*p* = 0.008) and in patients being irradiated with a radiation dose of more than 60 Gy (*p* = 0.003; Fig. 2a,b). In multivariate analysis none of the factors reached statistical significance.

### Patients without distant metastases at initial diagnosis

At a median follow-up of 7 months (range 1–86 months), OS and PFS at 12, 24 and 36 months were 51.3, 30.8 and 15.4% and 21.4, 14.3 and 14.3%, respectively. Median OS and PFS was 8 months (range 4–86 months) and 6 months (range 1–86 months), respectively.

Time from initial diagnosis to start of radiotherapy was 2.5 weeks (range 1–6 weeks).

**Fig. 3:**
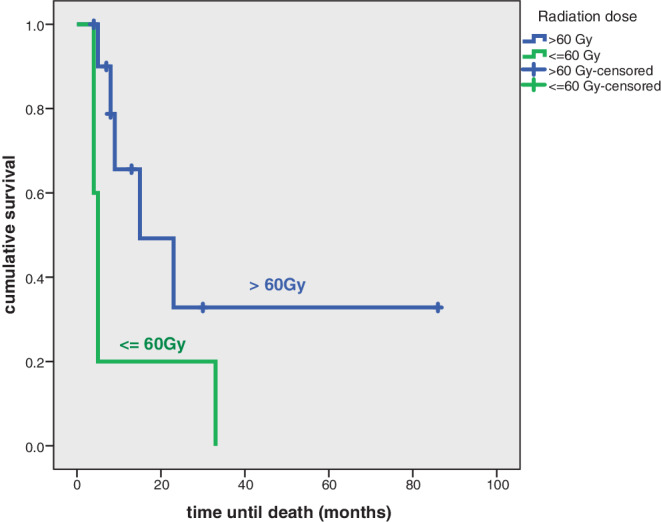
Cumulative overall survival depending on applied radiation dose in patients without distant metastases at first diagnosis

In univariate and multivariate statistical analysis female gender (*p* = 0.014) and radiation dose of more than 60 Gy (*p* = 0.05; Fig. 3) was significantly associated with an increased overall survival. In multivariate analysis none of the two factors reached statistical significance.

**Fig. 4:**
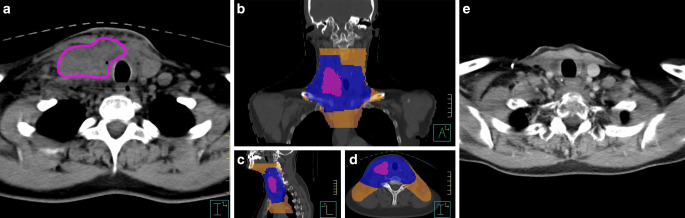
Patient 1 (Table 2). **a** Macroscopic residual tumor after surgery and before radiochemotherapy. **b** Planning target volume (*orange*): single fraction dose: 1.8 Gy. **c** Cumulative prescribed dose: 50.4 Gy and boost volume (*blue*): single fraction dose: 1.8 Gy. **d** Cumulative prescribed dose: 64.8 Gy, macroscopic residual tumor volume (*pink*). **e** Last follow-up computed tomography (CT) scan: no evidence of disease

Of the 16 patients without distant metastases undergoing radiotherapy 3 patients were alive and free from disease (follow-up time of these patients: 8, 30 and 86 months). See Table 2 and Fig. 4 for reference. Incidence of locoregional recurrence was 39.4% after 1 and 2 years. Only 1 patient had local failure, 7 patients had distant failure and 4 patients had locoregional and distant failure. One patient died from pneumonia secondary to an esophagotracheal fistula after RT and ongoing systemic therapy with lenvatinib. Locations of distant metastases were as follows: lung: *n* = 6, brain: *n* = 3, bone: *n* = 1, pleural, mediastinal and lung: *n* = 1.

**Table 2:** Data of patients being without evidence of disease after 2 years of follow-up

	Patient characteristics (gender, age at first diagnosis)	Tumor stage (TNM, UICC)	Therapy	Follow-up results
Patient 1	Female, 55 years	pT4b cN0 L0 V2 G4 R2, cM0	2/2013 Thyroidectomy, but macroscopic incomplete resection	86 months, no evidence of disease in 4/2020
		UICC stage IVB	3–5/2013 combined radiochemotherapy, radiotherapy: single fraction dose: 1.8 Gy, cumulative applied dose: 64.8 Gy	
			Cisplatin 20 mg/m^2^ BSA d1–5, d29–33	
Patient 2	Female, 29 years	pT3b, pN1a(8/25) R0 G3 Pn1 L1 V1 cM0, UICC stage IVB	2/2018: Thyroidectomy with complete tumor resection, 2–4/2018: combined radiochemotherapy: radiotherapy: single fraction dose: 2 Gy, cumulative applied dose: 66 Gy	30 months, no evidence of disease in 8/2020
			Cisplatin 20 mg/m^2^ BSA d1–5, d22–26, d43–47	

*BSA* body surface area

### Patients with distant metastases

At a median follow-up of 2 months (range 1–24 months), OS and PFS after 3, 6, 12 and 24 months was 38.1, 28.6, 19 and 9% and 18.8, 6.3, 0 and 0%, respectively. Median OS and PFS was 2.5 months (range 1–46 months) and 1 month (range 0–11 months).

Of the 16 patients, 2 had local failure and progressive distant metastases, 10 patients had distant progressive disease. Three patients died shortly after end of radiotherapy before the first follow-up visit. At the time of end of radiotherapy the primary tumor was controlled but disseminated pulmonary metastases were untreated and therefore it has to be assumed that patients died because of progressive distant metastases. One patient was lost to follow-up after prematurely stopping radiotherapy.

Time from first diagnosis to start RT was 2.5 weeks (range 0–20 weeks).

## Discussion

This monocentric analysis from a tertiary referral center shows high locoregional control rates of patients with ATC undergoing radio(chemo)therapy but overall survival is very low in accordance with the high incidence of distant metastases at initial diagnosis and on further follow-up.

Overall survival is 35.7 and 20.4% after 1 and 2 years for all patients and 51.3 and 30.8% after 1 and 2 years for patients without distant metastases at first diagnosis. These results are in line with other monocentric analyses of patients undergoing radio(chemo)therapy. Here overall survival rates after 1 year range from 20 to 48% [1, 9–12].

In a National Cancer database analysis [7] and also in a multicenter study [13], overall survival is lower than in our retrospective study. This might be because of the fact that in these database analyses all patients with ATC were included, even patients not undergoing radio- or radiochemotherapy. Database analysis showed that absence of lymph node and distant metastases, complete tumor resection, surgery of the primary tumor, high-dose radiotherapy of a minimum of 59.4 Gy [6] and radiochemotherapy as compared to radiotherapy alone [7] lead to improved overall survival. In other retrospective analyses surgery plus radiotherapy compared to surgery only [11], RT > 45 Gy [14] and any kind of chemotherapy [9] showed a significantly increased OS [15–17]. In monocentric analyses including only patients undergoing radiotherapy, the following factors were associated with an improved overall survival: surgery [12], radiotherapy > 60 Gy and trimodal therapy including surgery, radiotherapy and chemotherapy [1, 10]. A review including 17 retrospective studies [18] and also two SEER database analyses of 1567 [19] and 516 cases [20] reported that postoperative radiotherapy significantly decreased risk of death in all patients with resected ATC compared to surgery alone (*p* < 0.001). This study also showed that patients with stage IVA and B had a survival benefit from adjuvant (postoperative) radiotherapy compared to stage IV C patients.

In this analysis, overall survival in all patients significantly depends on UICC stage and radiation dose delivered. A UICC stage A and B and a radiation dose of > 60 Gy is associated with improved overall survival in univariate analysis. In multivariate analysis neither of the two factors reaches statistical significance. This might be because patients with distant metastases were treated with lower palliative radiation doses.

However, regarding only patients with UICC stage A and B radiation dose is still a significant predictor for improved overall survival. In the literature thresholds for the prognostic total RT dose are variably reported from 40 to > 60 Gy [1–3, 5, 6, 10, 12, 14]. In general, it has to be assumed that in patients without distant metastases, the radiation dose should be > 60 Gy (EQD2, alpha/beta = 10) to reach long-term local tumor control and in some patients even cure. However preclinical and clinical data suggest a benefit or rather a noninferiority for hypofractionated radiotherapy compared to normofractionated radiotherapy in ATC [21, 22]. In the palliative situation (stage IVC) a hypofractionated radiotherapy might be a time-sparing and effective treatment to reach local tumor control, on the one hand, and not to delay systemic treatment, on the other hand. Moreover, in addition to radiotherapy chemotherapy should be applied to increase the effect of radiotherapy. It should be emphasized that 3 of the patients included were free from disease at the last follow-up, all of them receiving cisplatin-based radiochemotherapy and a minimum radiation dose of 66 Gy. Because of the small size of the patient population, the addition of chemotherapy did not reach statistical significance; however, there was a trend for better overall survival by treating patients with combined radiochemotherapy (*p* = 0.06).

In the palliative setting the aim of radiotherapy is to prevent local symptoms like dyspnea and the need for tracheotomy. It is difficult to propose a certain radiotherapy concept for the palliative situation. Prescribed radiation dose and fractionation scheme should be adapted to the performance status and extent of distant metastases. In case of low tumor burden, good performance status and the possibility for targeted therapies a high-dose radiotherapy with a BED > 60 Gy should be applied to reach long-term locoregional control to prevent local symptoms and increase quality of life.

Locoregional control rate was 63.1% in the whole cohort of patients and 60.6% in patients without distant metastases at first diagnosis. Radio(chemo)therapy may therefore prevent local recurrence and the associated local symptom burden and tracheotomy in a high percentage of patients. However, despite the use of radio(chemo)therapy most patients died shortly after first diagnosis because of a rapid development of distant metastases. Standard chemotherapy regimens showed low response rates [23]; nevertheless, over the course of the last few years some aberrations, like BRAF mutation, RET translocation and NTRK fusion were detected that allow targeted systemic therapy. The combination of the BRAF inhibitor dabrafenib and the MEK inhibitor trametinib showed an overall response rate of 69% in patients with BRAF V600E-mutated anaplastic thyroid cancer [24]. The American Thyroid Association [25] recommends molecular testing of the following targets for which a targeted therapy is available: BRAF V600E mutation (present in 25–70% of all ATC), PD-L1 expression (expressed in 11–28% of all ATC), RET fusion (rare in ATC), ALK fusion (rare in ATC), NTRK fusion (rare in ATC). Moreover, there are about 15–40% Ras mutations, 65–75% TERT mutations and 50–70% TP 53 mutations in ATC but there is no targeted therapy available.

In patients without targetable aberrations, the multityrosine kinase inhibitor lenvatinib in combination with pembrolizumab is being tested [23, 26] and has shown promising response rates in a retrospective series of 6 patients with metastatic anaplastic thyroid cancer (66% complete responses, 16% stable disease and 16% progressive disease) [27].

Moreover in the future, radiologic and biologic markers like known from squamous cell carcinoma of the head and neck region may help to select patients for intensified first-line treatment strategies [28–31].

Limitations of the study exist due to the rarity of disease. As a result of the rarity of disease the assessment period of this study is very long leading to large inhomogeneity in treatment since radiation techniques and concepts as well as systemic treatment options will have varied over such a long period of time. BRAF mutational status was only examined in 11 of the 33 patients because this mutation was not known to be relevant for ATC at the beginning of the study. As such genetic findings like BRAF, RET or NTRK could not be correlated to survival rates in this analysis. Moreover, the small number of patients and the retrospective character of the study is a limitation, especially regarding statistical analysis with a small number of patients during a short follow-up.

Nonetheless, this monocentric analysis shows that in some patients long-term survival is reached using multimodal therapy including surgery, high-dose radiotherapy > 60 Gy and combined radiochemotherapy. However, because of the high rate of distant metastases there is a need for a better understanding of tumor biology and to integrate modern targeted therapies as soon as possible in the multimodal setting.

## Conclusion

Radiotherapy of more than 60 Gy leads to improved overall survival in patients with anaplastic thyroid cancer, but the total overall survival is short in this patient population because of a high incidence of distant metastases. In the future modern systemic treatment options should be integrated in multimodal therapy concepts.
